# Whole genome sequencing of recombinant viruses obtained from co-infection and superinfection of Vero cells with modified vaccinia virus ankara vectored influenza vaccine and a naturally occurring cowpox virus

**DOI:** 10.3389/fimmu.2024.1277447

**Published:** 2024-04-03

**Authors:** Diana Diaz-Cánova, Ugo Moens, Annika Brinkmann, Andreas Nitsche, Malachy Ifeanyi Okeke

**Affiliations:** ^1^ Molecular Inflammation Research Group, Department of Medical Biology, UiT - The Arctic University of Norway, Tromsø, Norway; ^2^ WHO Reference Laboratory for SARS-CoV-2 and WHO Collaborating Centre for Emerging Infections and Biological Threats, Robert Koch Institute, Berlin, Germany; ^3^ Section of Biomedical Sciences, Department of Natural and Environmental Sciences, School of Arts and Sciences, American University of Nigeria, Yola, Nigeria

**Keywords:** orthopoxvirus, poxvirus, smallpox, superinfection exclusion, recombination

## Abstract

Modified vaccinia virus Ankara (MVA) has been widely tested in clinical trials as recombinant vector vaccine against infectious diseases and cancers in humans and animals. However, one biosafety concern about the use of MVA vectored vaccine is the potential for MVA to recombine with naturally occurring orthopoxviruses in cells and hosts in which it multiplies poorly and, therefore, producing viruses with mosaic genomes with altered genetic and phenotypic properties. We previously conducted co-infection and superinfection experiments with MVA vectored influenza vaccine (MVA-HANP) and a feline Cowpox virus (CPXV-No-F1) in Vero cells (that were semi-permissive to MVA infection) and showed that recombination occurred in both co-infected and superinfected cells. In this study, we selected the putative recombinant viruses and performed genomic characterization of these viruses. Some putative recombinant viruses displayed plaque morphology distinct of that of the parental viruses. Our analysis demonstrated that they had mosaic genomes of different lengths. The recombinant viruses, with a genome more similar to MVA-HANP (>50%), rescued deleted and/or fragmented genes in MVA and gained new host ranges genes. Our analysis also revealed that some MVA-HANP contained a partially deleted transgene expression cassette and one recombinant virus contained part of the transgene expression cassette similar to that incomplete MVA-HANP. The recombination in co-infected and superinfected Vero cells resulted in recombinant viruses with unpredictable biological and genetic properties as well as recovery of delete/fragmented genes in MVA and transfer of the transgene into replication competent CPXV. These results are relevant to hazard characterization and risk assessment of MVA vectored biologicals.

## 1 Introduction

The *Orthopoxvirus* genus belongs to the *Poxviridae* family. The orthopoxviruses (OPXV) are viruses with large linear double stranded DNA genome (170 to 250kbp) (1). OPXV can infect vertebrates and insects (2). Among the OPXV that cause human diseases, *Variola virus* (VARV), vaccinia-like virus, *Cowpox virus* (CPXV) and *Monkeypox virus* (MPXV) are the most common (3–6). VARV is the causative agent of smallpox, a deadly viral disease, which was eradicated in 1980 as a result of a massive vaccination campaign (7). During the smallpox campaign, *Vaccinia virus* (VACV) was used as the smallpox vaccine and several VACV strains have been developed and used in different countries, such as New York City Board of Health (NYCBH) was used in the America, Tian tan in China, Ankara in Turkey, and Lister and modified vaccinia virus Ankara (MVA) in Europe (8–10).

MVA was administrated to over 120,000 people in Germany with no reported major side effects (10–13). MVA was derived from Chorioallantois vaccinia virus Ankara (CVA) by over 570 passages in primary chicken embryo fibroblasts (14). In this process, CVA genome suffered modifications including six large deletions and other mutations that lead to the reduction of the genome from 208kbp in CVA to 170kbp in MVA (15, 16). These mutations affected many genes involved in virus–host interaction and other genes responsible for evasion of the host immune response (16, 17).

As a result, MVA is unable to replicate productively in most mammalian cell lines (15, 17–21). Although some mammalian cell lines have been reported as permissive to MVA, such as Baby Hamster Kidney (BHK-21) cells (21, 22), and semi-permissive to MVA, such as Vero cells (African green monkey kidney epithelial cells) (15, 20). Recently, it was shown that a repair of the inactivated *C16R/B22R* in conjunction with the restoration of the deleted *C12L* gene restores production infection of many human cells with MVA (23). The host range restriction of MVA is considered the major biosafety advantage for its use as a vaccine vector along with its immunogenicity, its incapability to cause illness *in vivo* and its safety record (24–26).

Since the nineties, MVA has been widely tested in clinical trials as recombinant vector for vaccination against infectious diseases and cancers in both humans and animals (27, 28). Today, several MVA vaccines against HIV (29, 30), Ebola (31–34), respiratory syncytial virus (35), Middle East Respiratory Syndrome (36), cytomegalovirus (37), influenza (38, 39), tuberculosis (40) and malaria (41–43) are in different phases of clinical trials. MVA-BN (JYNNEUS or Imvanex) is licensed as a vaccine against Mpox and smallpox in both Europe and USA, and is currently being used for immunization against current global Mpox outbreak (44, 45). Even though MVA is already deployed as a vaccine and is a promising viral vector, there are still some biosafety aspects that should be considered during the biological hazard characterization of MVA and recombinant MVA (rMVA). One is the potential for MVA or rMVA to recombine with naturally occurring OPXV, which could lead to the rescue of interrupted or deleted genes in MVA or to transfer of the transgene to multiplication competent OPXV (46). Hence, the recombination could result in the emergence of novel mosaic viruses with atypical virulence and host range characteristics. Therefore, studies analyzing the potential of recombination between MVA and OPXVs are relevant to hazard characterization of MVA vectored vaccines. Co-infection and superinfection experiments constitute cell culture-based models to examine recombination of MVA with other OPXV during co-infection of semi-permissive cells as well as evaluate the possibility of superinfection exclusion in preventing recombination.

Recombination is not a rare event between OPXV. Natural occurring recombination events between OPXV have been reported (47–57). However, the possibility of recombination was considered negligible due to the low likelihood of co-localization of the viruses in the same cell, poor or no multiplication of MVA in many mammalian cells and superinfection exclusion (58, 59). The circulation of naturally zoonotic OPXV, such as CPXV and MPXV, has increased in recent years (6). Several cases of human cowpox infections caused by infected animals have been reported in Europe (49, 60–68) and the global outbreak of human Mpox has been reported in 110 countries (69) followed by increased vaccination with MVA. Therefore, the possibility of co-localization and recombination could have increased. We highlight that there is a need to better understand the mechanism of recombination and possible public health threat of recombination of two poxviruses after co- and superinfection.

In a previous study, we have demonstrated recombination in Vero cells co-infected and superinfected with recombinant MVA expressing the influenza virus *haemagglutinin* (*HA*) and *nucleoprotein* (*NP*) genes (MVA-HANP) and feline CPXV (27). In the present study, we sequenced the genome of putative recombinant viruses produced in semi-permissive Vero cells co-infected and superinfected with MVA-HANP and feline CPXV-No-F1, conducted genomic characterization of the parental and the putative recombinant viruses and mapped genome-wide recombination events in the recombinant viruses.

## 2 Materials and methods

### 2.1 Co-infection and superinfection of Vero cells

The co-infection and superinfection experiments with MVA-HANP and the naturally circulating Fennoscandian feline cowpox strain (CPXV-No-F1) were performed in Vero cells as previously described (27). The origin of the cowpox strain was described elsewhere (68). MVA-HANP was kindly provided by Dr. Bernard Moss, National Institutes of Health, USA. MVA-HANP contains the influenza virus *HA* (A/PR/8/34) and *NP* gene inserts (70). MVA-HANP was propagated in BHK-21 cells (ATCC CCL-10). CPXV-No-F1 and recombinant viruses were cultured on Vero cells (ATCC CCL-81). Vero cells and BHK-21 cells were propagated in minimal essential medium (MEM) supplemented with 10% fetal bovine serum (FBS). The cell cultures were maintained in a humidified 5% CO2 atmosphere at 37°C.

Vero cells are semi-permissive to MVA-HANP (15, 20) and permissive to CPXV (71). Vero cells were co-infected with MVA-HANP and CPXV-No-F1 at a multiplicity of infection (MOI) of 5 infection units per cell for each virus strain (**Supplementary Figure 1**). Superinfection with CPXV and MVA-HANP in Vero cells was performed in four experiments (**Supplementary Figure 1**). Vero cells were infected with CPXV-No-F1 at a MOI of 5. The infected Vero cells were superinfected with MVA-HANP at same MOI (of 5) after 4-hours (superinfection 1) or 6-hours post primary infection (ppi) (superinfection 3). The cells were incubated for 72 hours at 37°C. The same procedure was repeated for superinfection 2 and 4, but the primary infection was with MVA-HANP and the secondary infection with CPXV-No-F1 after 4-hours (superinfection 2) or 6-hours ppi (superinfection 4). After 72 hours ppi, the cells were harvested, freeze-thawn three times and sonicated. The co-infection and superinfection experiments were performed at the Poxvirus Laboratory (Biosafety Level-2), Department of Medical Biology, UiT - The Arctic University of Norway. The experiments were carried out at the appropriate containment levels.

### 2.2 Selection of putative recombinant viruses, plaque purification and immunostaining

The putative recombinant viruses were identified and selected in Vero Cells. The selection was based on (1) the expression of the Influenza virus HA protein and (2) plaque phenotype. The putative recombinant viruses that formed different plaque phenotype from the parental viruses were selected. The sonicated cells suspensions were inoculated on Vero cells and the viruses were plaque-purified several times before plaque amplification. The stock of the viruses was prepared from plaque purified viruses. The putative recombinant viruses carrying the influenza virus HA protein were detected by immunostaining, as described previously (22). Moreover, the plaque phenotype of the parental viruses was also examined in Vero cells.

### 2.3 Genome sequencing, genome assembly and annotation

Viral DNA of the plaque purified putative recombinant viruses and the parental virus MVA-HANP was isolated using QIAGEN Genomic-tip 100/G and QIAGEN Genomic DNA Buffer Set, following the manufacturer’s instructions (Qiagen, Hilden, Germany). The genomes were sequenced with Illumina MiSeq (Illumina Inc., San Diego, CA, United States) using reagent kit v3 with 2 × 300 bp paired-end reads and Oxford Nanopore Technology GridION (ONT; Oxford, United Kingdom), as previously described (49). Nanopore and Illumina library preparation have been described elsewhere (49). Nanopore sequencing was performed at the Genomics Support Centre Tromsø at UiT—The Arctic University of Norway and Illumina sequencing was conducted by the Norwegian Sequencing Centre, Oslo.

The genome assembly was performed using SPAdes v3.15.3 (72), as previously described (49). For the assembly of MVA-HANP, the parameter trusted-contigs and the reference genome MVA were used. The viral genomes were annotated using the Genome Annotation Transfer Utility (GATU) (73), as previously reported (49). Vaccinia virus Copenhagen (VACV-Cop), Choriollantois vaccinia virus Ankara (CVA) and MVA were used as reference genomes for the genome annotation of MVA-HANP. VACV strains were retrieved from the Viral Orthologous Clusters (VOCs) database (74). The parental viruses, CPXV-No-F1 (Genbank accession number OP125538) and MVA-HANP, were used as reference genomes for the genome annotation of the putative recombinant viruses. The complete genome of the parental CPXV-No-F1 has been published elsewhere (68).

### 2.4 Recombination analysis

The putative recombinant viral genomes were analyzed for possible recombination events using recombination detection program 4 (RPD4) (75) and SimPlot v3.5.1 (76) as described previously (49). The progeny virus genomes were aligned to the parental viruses and other CPXV strains (CPXV-Br and CPXV-Gri) with MAFFT v1.4.0 (77) implemented in Geneious Prime 2020.2.4. The CXPV strains were retrieved from the Viral Orthologous Clusters (VOCs) database (74). The gaps were not removed from the alignments. The recombination events identified by 5 of 7 methods (RDP (78), GENECONV (79), Bootscan (80), MaxChi (81), Chimaera (82), SiScan (83), and 3Seq (84)) with significant p-values (p ≤ 0.01) were considered potential recombinant events. Furthermore, the breakpoints in the recombinant genomes were checked manually in the alignments in case both programs could not detect the recombination event.

## 3 Results

### 3.1 Plaque phenotype formed by the putative recombinant viruses after co-infected and superinfected Vero cells

**Figure 1:**
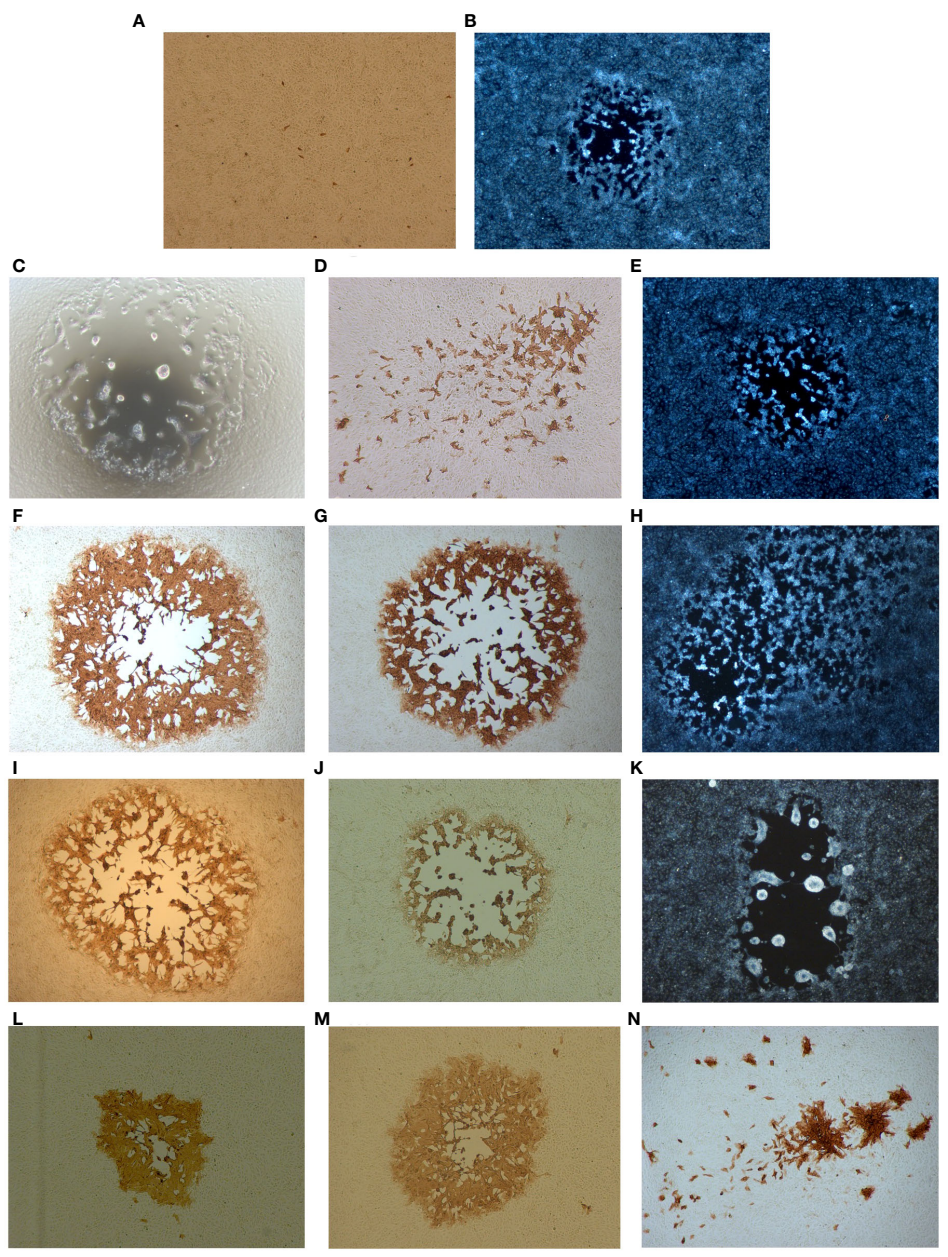
Plaque phenotypes of the parental viruses and the putative recombinant viruses. Confluent Vero cells were infected with the respective viruses and *HA* expression was monitored 48 h.p.i. by immunoperoxidase staining of fixed cells. **(A)** The parental virus MVA-HANP. **(B)** The parental virus CPXV-No-F1. **(C)** V-Rec1 from co-infection MVA-HANP and CPXV-No-F1. **(D, E)** V-Rec2 and V-Rec3 from superinfection 1 (primary infection with CPXV-No-F1 and secondary infection with MVA-HANP at 4h post primary infection, ppi). **(F–H)** V-Rec4, V-Rec5 and V-Rec6 from superinfection 2 (primary infection with MVA-HANP and secondary infection with CPXV-No-F1 at 4h ppi). (**I–M)** V-Rec7, V-Rec8, V-Rec9, V-Rec10 and V-Rec11 from superinfection 3 (primary infection with CPXV-No-F1 and secondary infection with MVA-HANP at 6h ppi). **(N)** V-Rec 12 from superinfection 4 (primary infection with MVA-HANP and secondary infection with CPXV-No-F1 at 6h ppi). All panels show representative fields at approximately 200× magnification.

The plaque phenotype caused by the putative recombinant viruses and the parental viruses were examined in Vero cells. The parental CPXV-No-F1 forms medium, semilytic plaques. Whereas MVA-HANP do not produce plaques; however, some Vero cells have positive immunostaining for the *HA* transgene as a result of MVA-HANP limited infection and expression of its *HA* transgene (**Figure 1**).

Different plaques phenotypes and transgene-expressing plaques were observed in co-infected Vero cells. One putative recombinant virus was isolated from co-infected Vero cells, which was named V-Rec1 (**Supplementary Table 1**). The plaque of this recombinant virus is large, lytic, and characterized by syncytium formation and cell lysis (**Figure 1C**). In addition, this recombinant forms *HA* transgene negative plaques.

Similarly, different plaque phenotypes were observed in superinfected Vero cells regardless the virus strain used for the primary infection and the timing (4 and 6 hours ppi). Additionally, both the transgene-expressing and non-transgene-expressing viruses display different plaque phenotypes (**Figures 1D–N**). Two, three, five and one viruses were selected from superinfection 1, superinfection 2, superinfection 3 and superinfection 4, respectively (**Supplementary Figure 1**). We referred to these putative recombinant viruses as V-Rec2, V-Rec3, V-Rec4, V-Rec5, V-Rec6, V-Rec7, V-Rec8, V-Rec9, V-Rec10, V-Rec11 and V-Rec12 (**Supplementary Table 1**).

V-Rec2 from the superinfection 1 expresses the *HA* transgene and produced small and non-lytic plaques with secondary spread (comet formation) (**Figure 1D**). Whereas V-Rec3 from the same experiment is transgene negative and forms medium and lytic plaques (**Figure 1E**). The plaques of V-Rec4 and V-Rec5 from superinfection 2 are large, lytic and transgene positive. Unlike V-Rec4 and V-Rec5, V-Rec6 is transgene negative and forms medium and semilytic plaques with extensive secondary spread (**Figures 1F–H**).

V-Rec7, V-Rec8 and V-Rec9 from superinfection 3 form large and lytic plaques (**Figures 1I–K**). However, compared to V-Rec7 and V-Rec8, V-Rec9 do not express the transgene and forms syncytia. The other two putative recombinant viruses were chosen from superinfection 3, the progeny virus V-Rec10 and V-Rec11. Both viruses express the *HA* transgene and form semilytic plaques (**Figures 1L, M**). However, the size of their plaques are different. V-Rec11 produces large plaques, whereas V-Rec10 generates medium plaques. V-Rec12 from superinfection 4 expresses the *HA* transgene and displays small and non-lytic plaques with comet formation (**Figure 1N**).

### 3.2 Different genome size of the putative recombinant viruses

**Figure 2:**
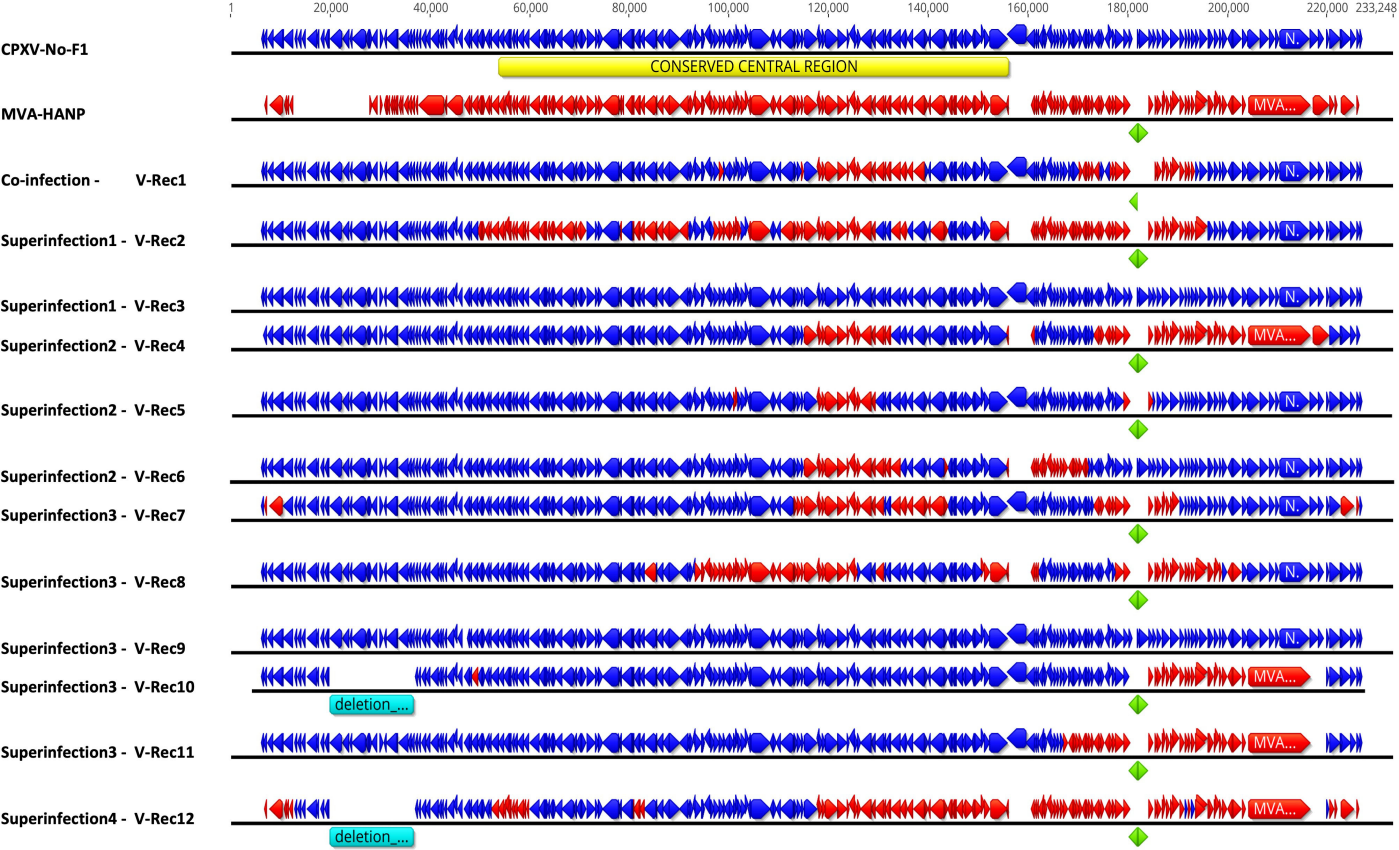
Genome map of the parental viruses (CPXV-No-F1 and MVA-HANP) and the putative recombinant viruses. The putative recombinant viruses were produced in Vero cells either co-infected or superinfected with CPXV-No-F1 and MVA-HANP. Superinfection 1, primary infection with CPXV-No-F1 and secondary infection with MVA-HANP at 4h post primary infection (ppi); Superinfection 2, primary infection with MVA-HANP and secondary infection with CPXV-No-F1 at 4h ppi; Superinfection 3, primary infection with CPXV-No-F1 and secondary infection with MVA-HANP at 6h ppi; Superinfection 4, primary infection with MVA-HANP and secondary infection with CPXV-No-F1 at 6h ppi. Blue blocks represent the coding sequences (CDS) from CPXV-No-F1. Red blocks represent CDS from MVA-HANP. Green blocks represent the influenza virus *hemagglutinin* (*HA*) and *nucleoprotein* (*NP*) transgenes. Turquoise blocks represent deleted regions in the recombinant viruses. Yellow block represents the conserved central region (*VACV-Cop F4L – VACV-Cop A24R*) in orthopoxvirus genomes.

The complete genomes of twelve putative recombinant viruses and the parental virus MVA-HANP were sequenced, assembled, and annotated (**Figure 2**). The whole genome sequences of MVA-HANP and V-Rec1 - V-Rec12 are available in GenBank, with Accession Number: OQ818667 and OQ822790 - OQ822801, respectively. The assembled genome of the parental MVA-HANP has a length of 181,712 bp, with inverted terminal repeats (ITR) of 9.8 kbp. Genome annotation predicted 199 coding sequences (CDS) in MVA-HANP genome (**Supplementary Table 2**). The sequencing analysis of the parental MVA-HANP showed that the double expression cassette containing the influenza *HA* and *NP* transgenes was inserted in *A51R/A55R* hybrid gene. However, the assembly of MVA-HANP showed that there are two populations of MVA-HANP, one with the complete double expression cassette (181,712 bp) and another with an incomplete double expression cassette (178,579 bp). A small part of *NP* transgene (~6%), whole *HA* transgene and downstream MVA-HANP flanking sequence (containing *VACV-Cop A56R* gene) are deleted in the latter. It only contains major part of the *NP* transgene (94%) and the upstream flanking MVA sequence.

The genome size and the number of predicted CDS of the parental and the progeny virus genomes are shown in the **Table 1** and **Supplementary Tables 2**, **3**. The length of progeny virus genomes was not uniform, ranging from 176.9 kbp to 221 kbp. Three putative recombinant viruses (V-Rec3, V-Rec5 and V-Rec9) have similar genome size to that of the parental CPXV-No-F1 and one recombinant virus (V-Rec12) possesses a smaller genome than that of the parental MVA-HANP (**Table 1**). The ITR of the progeny viruses ranged from 4.7 kbp to 8.3 kbp.

**Table 1:** Genome size and number of predicted CDS of CPXV-No-F1, MVA-HANP and the putative recombinant viruses.

Experiment		Virus	Genome (bp)	Inverted terminal repeat (bp)	CDS	Expressing *HA* transgene
		MVA-HANP	181,712	9882	199	Yes
		CPXV-No-F1	221,334	7929	217	Not Applicable
**Co-infection**	**CPXV-NO-F1/MVA-HANP**	V-Rec1	218,322	8219	216	No
**Superinfection**	**Superinfection 1** **(CPXV-NO-F1/MVA-HANP 4h)**	V-Rec2	215,275	8251	220	Yes
		V-Rec3	221,213	7929	217	No
	**Superinfection 2** **(MVA-HANP/CPXV-NO-F1-4h)**	V-Rec4	199,702	7045	210	Yes
		V-Rec5	220,926	7813	214	Yes
		V-Rec6	218,106	8365	217	No
	**Superinfection 3** **(CPXV-NO-F1/MVA-HANP-6h)**	V-Rec7	216,643	5908	213	Yes
		V-Rec8	216,086	8339	218	Yes
		V-Rec9	221,198	7853	217	No
		V-Rec10	185,955	6959	198	Yes
		V-Rec11	204,628	8054	212	Yes
	**Superinfection 4** **(MVA-HANP/CPXV-NO-F1-6h)**	V-Rec12	176,918	4694	197	Yes

The number of predicted CDS in the genomes of the putative recombinant viruses varied from 197 to 220 CDS (**Table 1**). The genomes of the putative recombinant viruses included more CDS than the parental MVA-HANP, with exception of V-Rec10 and V-Rec12. Furthermore, there were three viruses (V-Rec3, V-Rec6 and V-Rec9) that contained the same number of predicted CDS as the parental CPXV-No-F1.

### 3.3 Mosaic genome of the recombinant viruses

**Figure 3:**
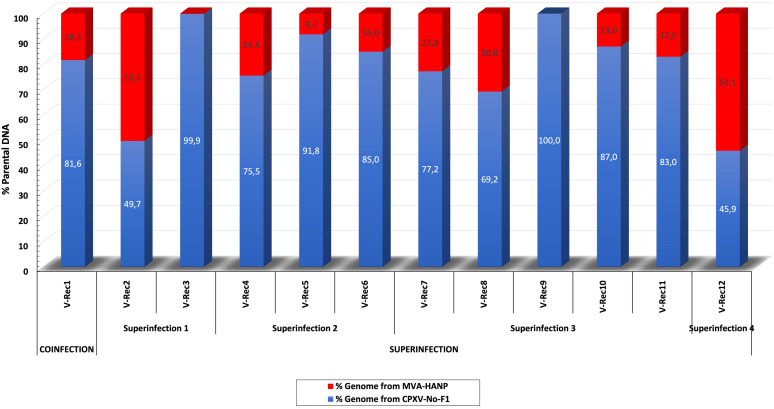
Percentage of DNA derived from the parental viruses (CPXV-No-F1 and MVA-HANP) in the putative recombinant viruses. The putative recombinant viruses were produced in Vero cells co-infected and superinfected with CPXV-No-F1 and MVA-HANP. Superinfection 1, primary infection with CPXV-No-F1 and secondary infection with MVA-HANP at 4h post primary infection (ppi); Superinfection 2, primary infection with MVA-HANP and secondary infection with CPXV-No-F1 at 4h ppi; Superinfection 3, primary infection with CPXV-No-F1 and secondary infection with MVA-HANP at 6h ppi; Superinfection 4, primary infection with MVA-HANP and secondary infection with CPXV-No-F1 at 6h ppi. Blue blocks represent the coding sequences (CDS) from CPXV-No-F1.

In order to localize the possible recombination events in the putative recombinant viruses, the twelve putative recombinant viruses were examined for recombination. The analysis confirmed recombination across the genome of eleven recombinant viruses (**Supplementary Table 4**). Only one virus, V-Rec9 from superinfection 3, does not show any recombination event based on the recombination analysis. The location of recombinant events in the genome of the progeny viruses are random, there are no recombination distribution pattern along their genomes. The recombination events occur both in the conserved central region (*VACV-Cop F4L* to *VACV-Cop A24L*) and the variable regions, including ITR (**Supplementary Table 5**; **Figure 2**). The number of recombinant breakpoints distribute in the central region varied from 0 to 16, whereas the number of breakpoints in the variable regions ranges from 1 to 6. The genomes of the recombinant viruses are a mosaic of the two parental strains. The lengths of the DNA segments exchanged between the parental viruses range from approximately 200 bp to 36 kbp (**Figure 2**). The percentage of DNA derived from the parental strains in the recombinant viruses is variable (**Figure 3**). The majority of the recombinant viruses have more DNA from the parental CPXV-No-F1 (∼45,9%-99,9%) than that from the parental MVA-HANP (∼0,1%-54,1%); nevertheless, some of them contain the *HA* and *NP* transgenes. Only two recombinant viruses, V-Rec2 and V-Rec12, contain more DNA from the parental MVA-HANP (**Figure 3**; **Supplementary Table 5**).

### 3.4 The influenza *HA* and *NP* transgenes are inserted in the same position in many of the recombinant viruses, but other genetic changes outside of recombination are present

**Figure 4:**
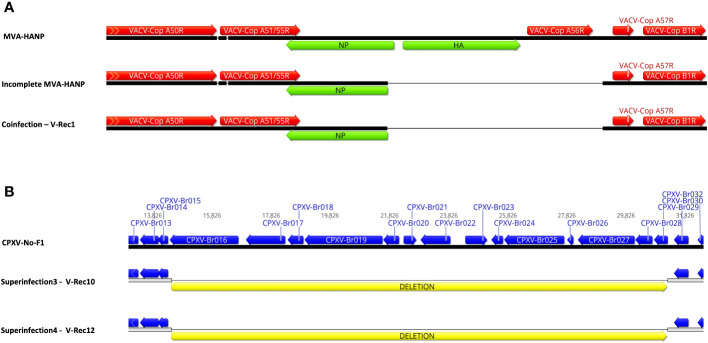
Comparison of the recombinant viruses with the parental virus. **(A)** Comparison of the recombinant region of V-Rec1 with MVA-HANP and incomplete MVA-HANP. V-Rec1 was produced in Vero cells co-infected with CPXV-No-F1 and MVA-HANP. Green blocks represent the influenza virus *hemagglutinin* (*HA*) and *nucleoprotein* (*NP*) transgenes. Red blocks represent the coding sequences (CDS) from MVA-HANP. **(B)** Deletion in V-Rec10 and V-Rec12. V-Rec10 was produced in Vero cells infected with CPXV-No-F1 and superinfected with MVA-HANP at 6h post primary infection (ppi) (superinfection 3). V-Rec12 was produced in Vero cells infected with MVA-HANP and superinfected with CPXV-No-F1 at 6h ppi (superinfection 4). Yellow blocks represent the deleted sequence in the progeny viruses.

The parental MVA-HANP harbored a double expression cassette containing the influenza *HA* and *NP* transgenes. The sequencing analysis of the eight *HA* transgene expressing recombinant viruses (V-Rec2, V-Rec4, V-Rec5, V-Rec7, V-Rec8, V-Rec10, V-Rec11 and V-Rec12) confirmed that these viruses contain an intact double expression cassette and its flanking sequences. The sequencing analysis of non-*HA*-transgene expressing recombinant viruses (V-Rec1, V-Rec3, and V-Rec6) revealed that the *HA-NP* insert is absent in V-Rec3 and V-Rec6, but not in V-Rec1. Interestingly, the latter (V-Rec1) contains part of the double expression cassette. It comprises major part of the *NP* transgene (94%) and the upstream flanking MVA sequence (**Figure 4A**) similar to the incomplete MVA-HANP. The presence of the incomplete MVA-HANP population with a partial deleted double cassette expression was corroborated by the occurrence of recombinant virus V-Rec1.

In addition to the recombination events, our analyses revealed that the eleven viruses underwent other genetic variations, such as deletions. Although most of them are located outside of the CDS regions (**Supplementary Table 6**). A large deletion of 16,761 bp is found close to the left terminal genomic region of V-Rec10 and V-Rec12 (**Figure 4B**). The deleted sequence comprises from *CXPV-BR016* gene to *CPXV-BR029* gene. Most of these genes encode proteins involve in host range. Additional small deletions and insertions are also detected within these two recombinant genomes (**Supplementary Table 6**). The other recombinant viruses also contain deletions, insertions and/or non-synonymous single-nucleotide mutations (nsSNMs). Interestingly, we found two nsSNMs in *VACV-Cop N2L* and *VACV-Cop K2L* genes of non-recombinant virus V-Rec9 (**Supplementary Table 6**). The nsSNM in the *VACV-Cop K2L* gene resulted in the introduction of a premature stop codon and consequently the truncation of the protein.

### 3.5 Rescue of loss genes and fragment/disrupted genes

As mentioned before there are two recombinant viruses with >50% of their genome derived from MVA-HANP. These two recombinant viruses rescued disrupted/deleted genes and, furthermore, gained genes that were absence in MVA-HANP. V-Rec12 rescued *CPXV-Br009, CPXV-Br032, CPXV-Br033, CPXV-Br034, CPXV-Br035* and *CPXV-Br036* genes (homologs to *VACV-Cop C16L, C5L, C4L, C3L, C2L* and *C1L* genes, respectively) that were lost in MVA during the attenuation process. Additionally, four fragmented/disrupted genes, *CPXV-Br037, CPXV-Br039, CPXV-Br043* and *CPXV-Br078* (homologs to *VACV-Cop N1L, K1L, K5L* and *O1L* genes, respectively), were rescued in the recombinant virus V-Rec12 by recombination. Although *O1L* gene is also fragmented in VACV-CVA. Moreover, the recombinant virus gained seven more genes that were absent in MVA and VACV-CVA strains: *CPXV-Br010, CPXV-Br011, CPXV-Br012, CPXV-Br013, CPXV-Br014, CXPV-Br015* genes and one gene (*No-F1-009* gene) that encodes BTB Kelch-domain containing protein. Compared to V-Rec12, V-Rec2 gained six additional genes (*CPXV-Br002, CPXV-Br016, CPXV-Br017, CPXV-Br018, CPXV-Br19* and *CPXV-Br020* genes) by recombination. Furthermore, the recombination events in V-Rec2 rescued deleted genes: *CPXV-Br005, CPXV-Br009, CPXV-Br032, CPXV-Br033, CPXV-Br034, CPXV-Br035, CPXV-Br036, CPXV-Br039, CPXV-Br040, CPXV-Br213, CPXV-Br217* and *CPXV-Br226* genes (homologs to *VACV-Cop B28R, C16L, C5L, C4L, C3L, C2L, C1L, M1L, M2L, B20R, C12L* and *C22L* genes). Additionally, twelve fragmented/disrupted genes in MVA were also rescued in this recombinant virus (*CPXV-Br003, CPXV-Br006, CPXV-Br008, CPXV-Br023, CPXV-Br025, CPXV-Br027, CPXV-Br041, CPXV-Br078, CPXV-Br207, CPXV-Br212, CXPV-Br223* and *CPXV-Br227* genes).

## 4 Discussion

In our previous study, we performed co-infection and superinfection experiments with a naturally circulating Fennoscandian CPXV-No-F1 and MVA carrying an influenza *HA* and *NP* transgene in semi-permissive Vero cells. We have showed that recombination between these viruses occurred in both co-infected and superinfected Vero cells. Although the likelihood of recombination in co-infected and superinfected Vero cells was always considered low because (1) Vero cells are semi-permissive to MVA (15, 20) and (2) mechanisms as superinfection exclusion prevent the superinfection of the infected cell with a second virus (58, 59). A previous study has reported recombination between human CPXV (hCPXV) and MVA-HANP in co-infected BHK-21 cells (22). Compared to Vero cells, those cells are fully permissive to MVA-HANP and CPXV (20). Viral DNA replication in non-permissive cells to MVA infection is not blocked (85), therefore the likelihood of recombination increases since recombination and viral DNA replication are connected (86, 87). In our superinfection experiments, the time gap between the first and the second infection was 4h and 6h ppi to ensure the establishment of primary infection since it has been reported that VACV DNA synthesis in HeLa cells starts 2 hours postinfection (88). Although the viral DNA synthesis of the primary virus is not needed for superinfection exclusion (59, 89). A study demonstrated the time of superinfection of 4 and 6 hours after primary infection with VACV produced 90% and 99% exclusion of superinfecting virus, respectively (89).

The recombination between MVA-HANP and CPXV-No-F1 generated mosaic genomes containing genomic material from both parental viruses. Several progeny viruses displayed plaque morphologies different from that of the parental viruses. This was also observed in the progeny viruses arising from superinfected Vero cells with MVA-HANP and CPXV-No-F1, using 2h ppi (27). Similarly, the recombinant viruses from BHK-21 cells co-infected with MVA-HANP and hCPXV displayed non-parental and parental traits with respect to plaque phenotype, *in vitro* host range and cytopathogenicity (22, 71).

Our recombinant viruses showed different plaque phenotypes. It has been reported that the proteins encoded by *VACV-Cop F5L, F11L, F12L, F13L*, *A33R*, *A34R*, A36R, *A56R*, and *B5R* genes may be involved in determining the plaque morphology (90–95). Some of these genes were fragmented (i.e. *VACV-Cop F5L* and *VACV-Cop F11L* gene) or encompassed some deletions (i.e. *VACV-Cop A36R*) in MVA (16). Two of our recombinant viruses (V-Rec2 and V-Rec12) formed small and non-lytic plaques. These recombinant viruses contained the defective *VACV-Cop F5L, F11L* and *VACV-Cop A36R* genes from MVA-HANP. F5L and F11L proteins are required to form normal plaques. They increase the plaque size and only F5L protein promotes the formation of central plaque clearing (90–92). The deletion of the gene encoding A36R protein decreased the plaque size (96). Additionally, these two recombinant viruses displayed plaques with comet formation, similar to those of the recombinant virus V-Rec6. The genes associated to the formation of comet-shaped plaques are *VACV-Cop A33R, A34R* and *B5R* (93, 94, 97). Those recombinant viruses contained *VACV-Cop A33R, A34R* and *B5R* genes from MVA-HANP, except for recombinant virus V-Rec6 that contained *VACV-Cop B5R* gene derived from CPXV-No-F1. Other recombinant viruses that only have *VACV-Cop B5R* gene from MVA-HANP, as well as *VACV-Cop A33R* and *VACV-Cop A34R* genes from CPXV-No-F1, did not show plaques with comet formation.

Only two viruses produced syncytial plaques: recombinant virus V-Rec1 and non-recombinant V-Rec9. The latter produces a truncated K2L protein due to the introduction of an earlier stop codon in the *VACV-Cop K2L* gene as a result of a nsSNM. It has been reported that the lack of K2L protein causes the fusion of infected cells (98–100). This protein forms a complex with A56 protein, and the heterodimer prevents syncytia formation (101) (98–100, 102). The *VACV-Cop A56R* gene of V-Rec9 was intact; in contrast, this gene was deleted in the recombinant virus V-Rec1.

Several recombinant viruses were transgene positive. The proportion of HA-expressing recombinant viruses in the co-infected and superinfected Vero cells were reported elsewhere (27). The *HA*-expressing recombinant viruses had the complete double expression cassette, while the non-HA-expressing recombinant virus V-Rec1 contained an incomplete expression cassette without the *HA* transgene similar to the incomplete MVA-HANP. It seems that this progeny virus was the result of recombination of CPXV-No-F1 and the incomplete MVA-HANP. The instability of the transgene in MVA-HANP as well as in recombinant progeny viruses from BHK-21 cells co-infected with MVA-HANP and CPXV-No-H1 has been previously reported. MVA-HANP and the recombinant viruses were genetically unstable and lost the transgene during cell culture passages (22, 71). The instability of the transgene is one of the major concerns in the production of viral vector vaccines because any mutation in the expression cassette could lead to unpredicted characteristics. Furthermore, the transgene is used as a tag to monitor the expression cassette in the recombinant progeny viruses. Therefore, the loss or partial loss of the transgene hinders the tracking of released recombinant progeny viruses. Another concern about the use of MVA-HANP is to transfer the transgene into a multiplication competent OPXV (46). In this study, we have shown that the transgenes were transferred to the recombinant viruses with a genome mainly derived from CPXV-No-F1. Furthermore, the recombinant progeny viruses with the transgene displayed new and non-parental plaque phenotypes. A biological characterization of the recombinant viruses is required to investigate their host range, multiplication curves at low and high moi, cell tropism, transmissibility and virulence.

Compared to CVA, MVA had lost several genes and 25 genes were fragmented and/or suffered mutations during the attenuation process, such as the host range genes *VACV-Cop K1L* and *C12L* (16, 18). The recombination of MVA with a multiplication competent OPXV may lead to the restoration of disrupted/deleted genes in MVA. In this study, we observed that two of our recombinant viruses, that had >50% DNA from MVA-HANP, rescued deleted and fragment host range genes, such *CXPV-Br009* (*VACV-Cop C16L*) and *CPXV-Br041* (*VACV-cop K1L*) (23, 103). Even these recombinant viruses gained new host range genes, *crmB/CPXV-Br226* (*VACV-Cop B28R*) and *vCD30/CPXV015* (103, 104). The recombination of MVA with a wild OPXV was considered negligible since smallpox was eradicated. However, the circulation of the naturally OPXV (3, 49, 68, 105–107) and the emergence of new OPXV in the last few years (50, 108, 109) have increased the likelihood of recombination between MVA and a naturally replication competent OPXV during co-infection or superinfection of the same cell or host. For instance, the ongoing global outbreak of Mpox and prophylactic or post-exposure vaccination with MVA-BN provide a good scenario for co-infection/superinfection and subsequent recombination between MVA and MPXV. In addition, several orthopoxvirus outbreaks in humans have been reported worldwide (6, 110). Our study has shown that insertion of the transgene into the genome of a co-infecting or superinfecting OPXV was specific, but recombination in other parts of the genome were nonspecific and unpredictable. In addition, some of the recombinant viruses lost some or part of the transgenic cassette even when they were inserted into the intended genomic regions, and the loss of transgene may preclude tracking of recombinant viruses. To evaluate the potential for recombination and robust monitoring of potential recombinant viruses, hazard characterization and risk assessment of MVA vectored biologicals should include genome wide characterization.

Finally, it is important to highlight the limitations of this study. First, the high MOI used in our co-infection and superinfection experiments may not be achievable under natural co-infection/superinfection. Second, our experiments were done in cell cultures and an extrapolation cannot be made to animals with an intact immune system Vero cells in particular is a continuous cell line lacking type 1 interferon gene although it still has interferon receptors (111). Third, our use of selection markers like plaque phenotype, expression or non-expression of the transgene may have introduced a selection bias and thus underestimate the pattern and diversity of recombination between co-infecting or superinfecting viruses. Fourth, the likelihood of recombination in co-infected and superinfected cells is low; however, the possibility of co-localization has raised because the circulation of naturally zoonotic OPXV (e.g. CPXV and MPXV) has increased in recent years as well as the vaccination with MVA. Fifth, this study did not calculate recombination frequency (RF) with respect to the transgene cassette (112), and the number of recombinant viruses selected was low to statistically infer genome-wide patterns and diversity of recombination between CPXV and MVA-vectored vaccine during co-infecting and superinfecting scenarios. Future studies will address these limitations through plaque independent deep sequencing analysis of cell cultures and animals co-infected or superinfected with MVA and CPXV under conditions that better reflects natural infection.

In conclusion, superinfection exclusion and low permissivity of Vero cells to MVA did not prevent the recombination between MVA vectored vaccines and the naturally circulating CPXV during superinfection of cells. The recombination between MVA-HANP and the naturally circulating CPXV-No-F1 in co-infected and superinfected Vero cells lead to the generation of progeny viruses with non-parental biological and genetic characteristic as well as the regaining of delete/fragmented genes in MVA-HANP, transfer of the transgene into CPXV and introgression of other MVA genes to CPXV. This is a proof-of-concept study and future studies will examine the risk of recombination between MVA or rMVA and naturally circulating OPXV during co-infection and superinfection by determining the likelihood of recombination and its consequences.

## Data availability statement

The datasets presented in this study are deposited in the Genbank (https://www.ncbi.nlm.nih.gov/genbank/), accession numbers can be found below: OQ818667, OQ822790, OQ822791, OQ822792, OQ822793, OQ822794, OQ822795, OQ822796, OQ822797, OQ822798, OQ822799, OQ822800 and OQ822801.

## Author contributions

DD: Conceptualization, Data curation, Formal analysis, Investigation, Methodology, Writing – original draft, Writing – review & editing. UM: Conceptualization, Formal analysis, Funding acquisition, Investigation, Methodology, Project administration, Resources, Supervision, Writing – original draft, Writing – review & editing. AB: Data curation, Formal analysis, Investigation, Methodology, Writing – review & editing. AN: Data curation, Formal analysis, Investigation, Methodology, Writing – review & editing. MO: Conceptualization, Formal analysis, Funding acquisition, Investigation, Methodology, Project administration, Resources, Supervision, Writing – original draft, Writing – review & editing.
