# Supplementary material for: Whole genome sequencing of recombinant viruses obtained from co-infection and superinfection of Vero cells with modified vaccinia virus ankara vectored influenza vaccine and a naturally occurring cowpox virus
Source: Front Immunol. 2024 Apr 3;15:1277447. doi: 10.3389/fimmu.2024.1277447 (PMC11021749; doi:10.3389/fimmu.2024.1277447)
Supplement: Supplementary file 1 [file DataSheet_1.docx]

Supplementary Material

Whole genome sequencing of recombinant viruses obtained from co-infection and superinfection of Vero cells with Modified Vaccinia virus Ankara vectored influenza vaccine and a naturally occurring cowpox virus

[**Diana Diaz-Cánova**](https://www.frontiersin.org/people/u/1663502)**^1^,**[**Ugo Moens**](https://www.frontiersin.org/people/u/716562)**^1^*,**[**Annika Brinkmann**](https://www.frontiersin.org/people/u/1197063)**^2^, Andreas Nitsche^2^ and**[**Malachy Ifeanyi Okeke**](https://www.frontiersin.org/people/u/1746973)**^3^***

*** Correspondence:** Ugo Moens, [ugo.moens@uit.no](mailto:ugo.moens@uit.no); Malachy Ifeanyi Okeke, [malachy.okeke@aun.edu.ng](mailto:malachy.okeke@aun.edu.ng)

# Supplementary Figures


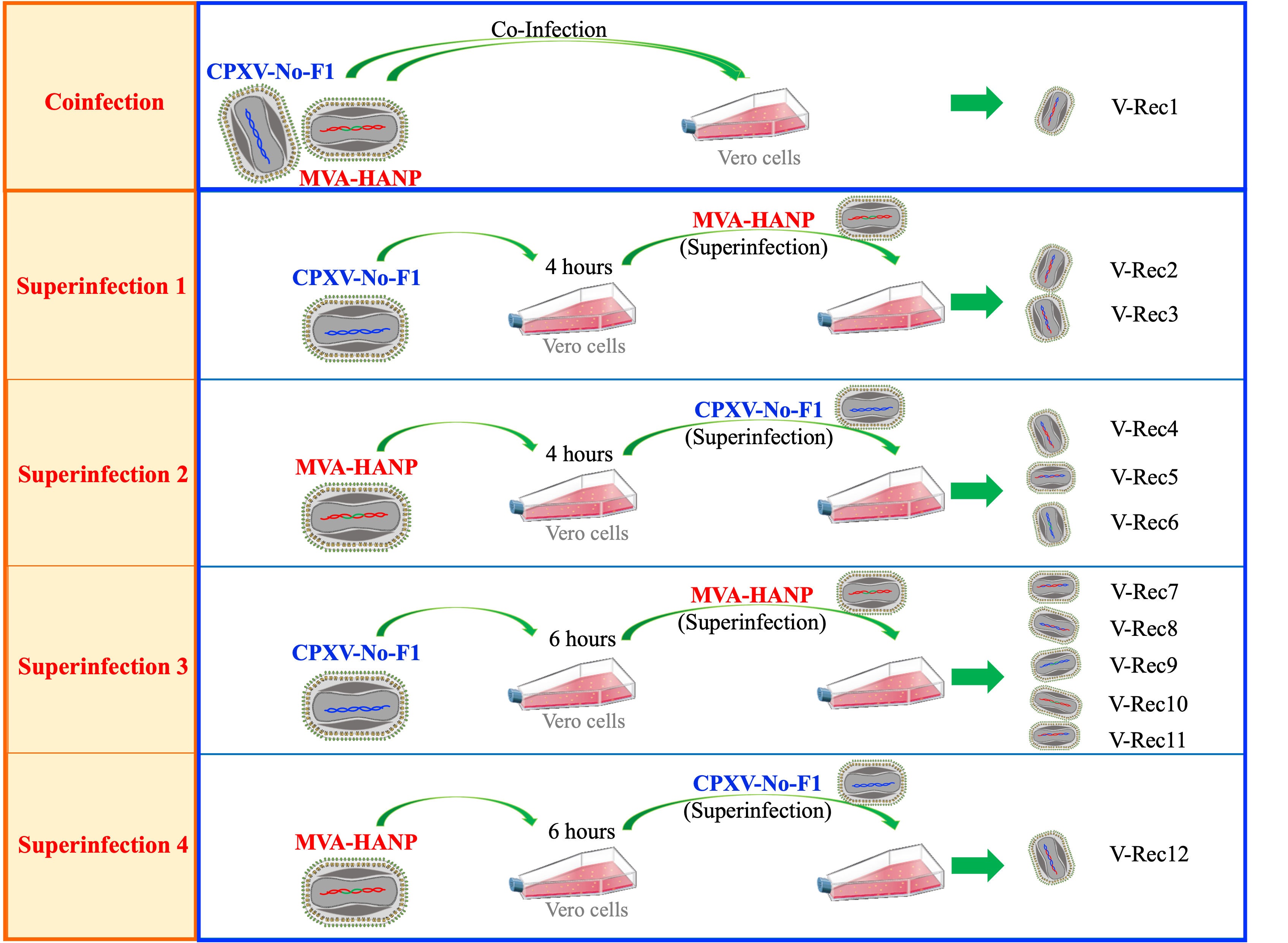


**Supplementary Figure S1.** Schematic representation of the coinfection and superinfection experiments using MVA-HANP and *Cowpox virus* (CPXV-No-F1).
